# A Novel Real-time Phase Prediction Network in EEG Rhythm

**DOI:** 10.1007/s12264-024-01321-z

**Published:** 2024-11-29

**Authors:** Hao Liu, Zihui Qi, Yihang Wang, Zhengyi Yang, Lingzhong Fan, Nianming Zuo, Tianzi Jiang

**Affiliations:** 1 School of Artificial Intelligence, University of Chinese Academy of Sciences, Beijing, 100049 China; 2 Brainnetome Center, Institute of Automation, Chinese Academy of Sciences, Beijing, 100190 China; 3 University of Chinese Academy of Sciences, Beijing, 100049 China; 4 Xiaoxiang Institute for Brain Health and Yongzhou Central Hospital, Yongzhou, 425000 China

**Keywords:** Real-time EEG phase prediction, Closed-loop neuromodulation, EEG phase-triggered regulation, EEG rhythm, TMS-EEG co-registration

## Abstract

Closed-loop neuromodulation, especially using the phase of the electroencephalography (EEG) rhythm to assess the real-time brain state and optimize the brain stimulation process, is becoming a hot research topic. Because the EEG signal is non-stationary, the commonly used EEG phase-based prediction methods have large variances, which may reduce the accuracy of the phase prediction. In this study, we proposed a machine learning-based EEG phase prediction network, which we call EEG phase prediction network (EPN), to capture the overall rhythm distribution pattern of subjects and map the instantaneous phase directly from the narrow-band EEG data. We verified the performance of EPN on pre-recorded data, simulated EEG data, and a real-time experiment. Compared with widely used state-of-the-art models (optimized multi-layer filter architecture, auto-regress, and educated temporal prediction), EPN achieved the lowest variance and the greatest accuracy. Thus, the EPN model will provide broader applications for EEG phase-based closed-loop neuromodulation.

**Supplementary Information:**

The online version contains supplementary material available at 10.1007/s12264-024-01321-z.

## Introduction

Electroencephalography (EEG) is a non-invasive technique for recording the electrical activity of neurons in the brain [1, 2]. EEG rhythms are classified into delta, theta, alpha, beta, and gamma signals according to the period of oscillation [3]. The power and the phase information of these rhythms have been shown to evaluate cognitive processing in single or multiple brain regions [4–8]. Integrating the characteristics of EEG signals to achieve real-time control of external stimuli and form closed-loop neuromodulation strategies has been attracting increasing attention. The real-time EEG phase has been used to drive external visual stimuli and effectively regulate the frequency and power of the alpha rhythm (8 Hz–12 Hz) in a novel neurofeedback system [9]. Moreover, the phase of the EEG rhythm can also serve as a switch signal to optimize and control the regulation of external neuromodulation devices such as transcranial magnetic stimulation (TMS).

TMS is one of the most widely used and powerful tools for non-invasive brain stimulation and is commonly used to treat physical and psychological disorders as well as to study and modulate cognitive behavior [10–14]. It has been shown to safely and reliably perturb the cerebral cortex, while different stimulation sessions have been shown to excite and inhibit specific brain areas [15–18]. Currently, the most widely used TMS modulation techniques are still open-loop, i.e., the stimulus parameters are fixed and not optimally modulated in real time based on the subject’s brain state. Open-loop TMS has limited applications, poor treatment stability, and obvious individual differences [19–22]. Therefore, understanding how to evaluate the real-time state of the brain and dynamically adjust the TMS stimulation parameters, changing them from "one size fits all" to accurate closed-loop neuromodulation, is still under investigation [23–26].

Several research teams have tried to use the phase of real-time EEG to control the modulation of TMS at the motor hand area to understand how a real-time EEG signal shapes corticomotor excitability, as measured by the amplitude of the motor evoked potential (MEP); however, the results have been inconclusive [27–31]. In real-time EEG phase-based TMS closed-loop modulation, only a limited narrow-band EEG signal, such as a window of 500 ms before the TMS trigger (time 0), can be processed [27]. Given the unavailability of future EEG samples, the application of a causal filter becomes imperative for the conventional phase prediction framework to isolate the desired EEG rhythm, as its output is contingent upon present and past inputs exclusively [32]. However, a causal filter can also result in phase delay and have a significant impact on phase prediction, as the beginning and end of this narrow EEG signal are severely distorted. The instantaneous point at the end of the narrowed data usually needs to be discarded and refitted or linearly interpolated based on the phase of the unaffected data segment.

Currently, many phase prediction frameworks are widely used to restore the instantaneous phase, such as optimized multi-layer filter architecture (MLOF) [33], auto-regress (AR) [27], and educated temporal prediction (ETP) [34]. MLOF is a machine learning-based approach that combines two fully connected layers and a Softmax operation to predict the instantaneous phase from raw EEG data. The AR and ETP models apply conventional causal filters to isolate the targeted EEG rhythm and subsequently predict the real-time phase at the distorted edge. A notable distinction lies between the two methodologies: the AR model refits out the EEG rhythm signal affected by the filter through polynomial fitting, followed by Hilbert transformation for real-time phase calculation. Conversely, the ETP model extracts the peak phase of the unaffected rhythm, interpolates a real-time phase at time 0, and applies statistical learning techniques to mitigate interpolation errors. Although the circular mean value of the phase prediction error in the alpha band is small, the variance is usually large [34]. To reduce the variance of the phase prediction, subjects and trial times are usually pre-selected, such as the AR model requires the alpha power to be at least 25% of the total power [27].

**Fig. 1:**
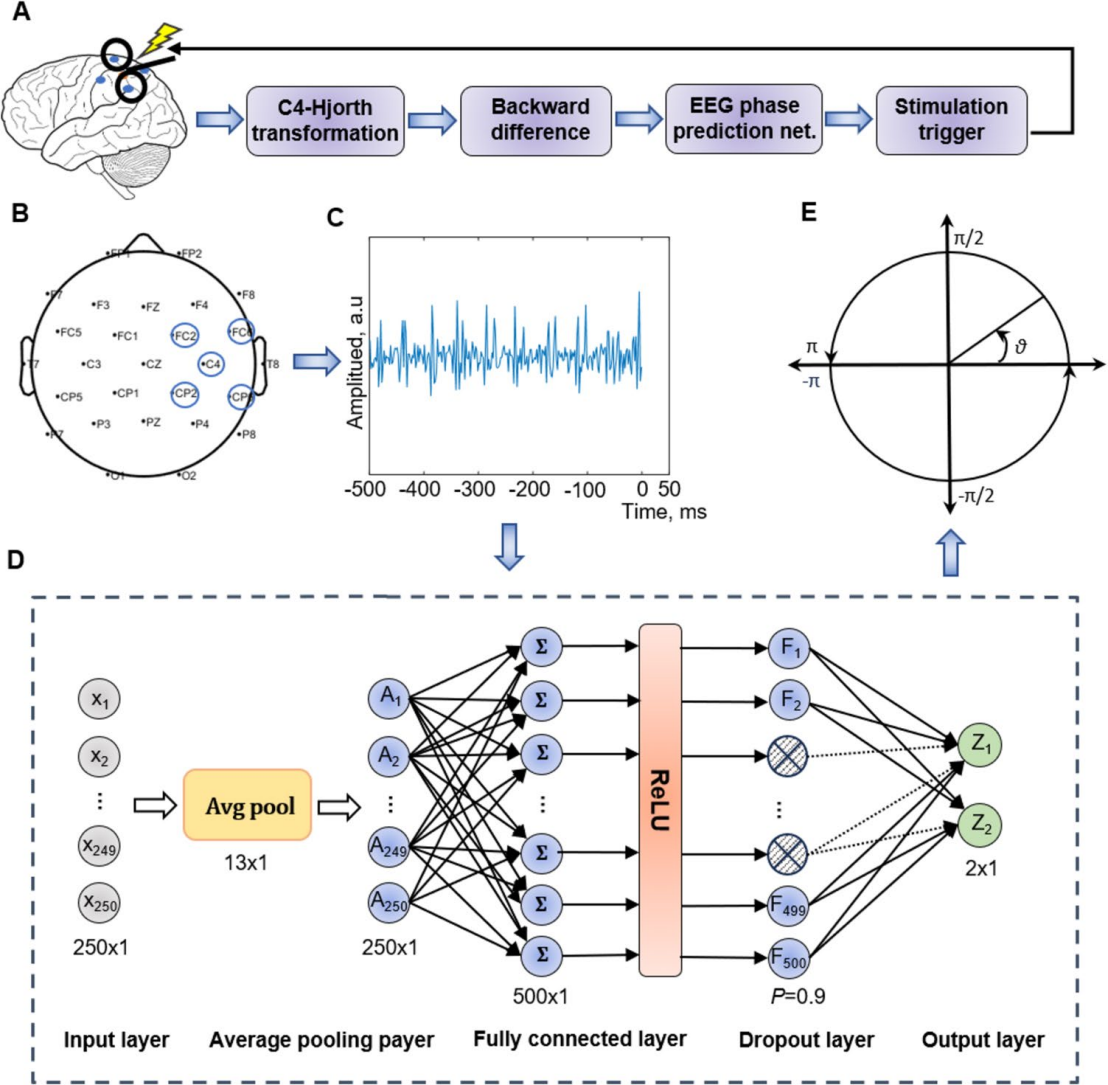
The phase prediction pipeline of an EEG μ rhythm using the EEG phase prediction network (EPN). **A** Flowchart of the phase prediction. The process mainly consists of using a Hjorth transform to extract the local μ-rhythm, applying the backward difference to remove large electrode drifts, using the EPN model, and sending a stimulation event to operate the TMS machine. **B** The C4-Hjorth transform. C4 is the centered electrode, while FC2, FC6, CP2, and CP6 are the surrounding electrodes. **C** Backward difference. The output $y\left( n \right){ } = { }x\left( {n + 1} \right) - x\left( n \right)$, $x\left( n \right)$ is the signal at time index $n$. **D** Architecture of the EPN model consists of 5 layers: the input layer (250 × 1), the average pooling layer (13 × 1), the fully connected layer (500 × 1), the dropout layer ($P{ } = { }0.9$), and the output layer (2 × 1). The dashed nodes and dotted lines of the dropout layer illustrate neurons being dropped during model training. **E** Stimulation trigger. The process checks the output phase $\vartheta$ of the EPN model, filters the targeted phase of interest, and sends a stimulation event to start a single-pulse session.

EEG signals are temporal and non-stationary, approximating them as steady-state may introduce prediction errors. In the field of time series forecasting, there are various machine learning models used to predict time series signals such as transformer [35], long-term time series forecasting linear (LTSF-Linear) [36], recurrent neural network (RNN) [37], etc. We propose that combining conventional filtering with machine learning methods can effectively capture non-linear EEG features, thereby reducing phase prediction variance and increasing prediction accuracy. Therefore, we developed a novel EEG phase prediction network (EPN) shown in Fig. 1 and validated the performance using pre-collected EEG data, simulated EEG data, and real-time experimental data. The results confirmed that the EPN achieved the greatest prediction accuracy and the lowest prediction variance compared with MLOF, AR, and ETP without preselected subjects or stimulation trials. The purpose of this study was to develop a method that could be used for both offline and real-time EEG phase prediction.

## Materials and Methods

### Phase Prediction Frameworks

Optimizing the TMS strategy based on the instantaneous EEG phase is a hot area in closed-loop neuromodulation, which needs to be balanced between accuracy and speed. In real-time situations, only narrow-band EEG signals can be acquired before stimulation; this leads to the instantaneous phase being distorted by the causal filter. There are several models for restoring the instantaneous EEG phase.

### Optimized Multi-layer Filter Architecture (MLOF)

The key feature of this method is to combine two fully connected (FC) layers, one of which uses a Softmax operation, *via* a dot-multiplying operation to capture multiple frequency representations and be able to gate between them. The machine learning architecture is designed to make a causal mapping from raw EEG data to the instantaneous phase. A detailed description can be found in [33].

### Auto-regress Model (AR)

Since the edge of a narrow-band EEG signal is often distorted by a causal filter, the AR model regresses out the points so that the phase information can be subsequently calculated by the Hilbert transform in a single filtered EEG data trial [27, 38].

The EEG rhythm data can be fitted using Equation (1):

1 $$ X_{t} { } = { }a_{1} X_{t - 1} + a_{2} X_{t - 2} + \ldots + a_{p} X_{t - p} + \varepsilon_{t} $$

where $X_{t}$ is the filtered EEG rhythm data at time *t*, $p$ is the order, $\varepsilon_{t}$ is the error at time *t*, and $a$ is the factor of the AR model. The factor $a$ is usually calculated using the Yule-Walker method and is updated every time new data are collected. The order *p* is usually set empirically.

### Educated Temporal Prediction (ETP)

The distorted instantaneous phase can be linearly interpolated by using the points that are unaffected or little affected by the causal filter in the narrow EEG signal. For example, ETP calculates the frequency cycle of the individual alpha frequency (IAF) specific to each subject based on the pre-acquired resting EEG data [34]. Then the peaks are extracted from the filtered narrow-band rhythm and treated as the 0-degree Hilbert phase. The mean absolute phase bias of the peaks to the ground truth phases is then calibrated through statistical learning and added into the IAF cycle. Finally, the target phrase of interest can be obtained by interpolation based on the nearest peak location according to Equation (2).

2 $$ T_{{{\text{adj}}^{*} }} = \frac{{\theta \times T_{{{\text{adj}}}} }}{{2{\uppi }}} $$

where $T_{{{\text{adj}}^{*} }}$ is the new value of the individual alpha frequency cycle and is added to the last detected alpha peak. $T_{{{\text{adj}}}}$ is the IAF cycle learned and bias-adjusted from the pre-recorded resting EEG data. $\theta { }$ is the target phase frequency for TMS stimulation.

### EEG Phase Prediction Network (EPN)

EEG is non-stationary, so approximation to a steady-state signal introduces prediction errors, and a cross-trial approach is needed to learn the rhythmic characteristics of narrow-band EEG signals. We thought that combining conventional filtering with machine learning could significantly reduce the phase prediction variance and increase the prediction accuracy. Therefore, we developed the EPN model, which can detect the non-linear EEG features and directly predict the instantaneous EEG phase.

As shown in Fig. 1D, the EPN consists of five layers: Input layer, Average pooling layer, Fully connected layer, Dropout layer, and Output layer.

*1. Input Layer*

This layer receives the input EEG data with a sampling window of 250 (500 ms at a sampling rate of 500 Hz). Its function is to pass the input data to the subsequent layers for processing.

*2. Average Pooling Layer (Avg Pool)*

The average pooling layer function is similar to a moving average filter for time series data, suppressing noise and enhancing the robustness of the model. For a given EEG sequence $$ x$$, the output of the average pooling layer is:

3 $$ A\left( n \right){ } = { }\frac{1}{s} \mathop \sum \limits_{{i{ } = { }n}}^{n + s} x\left( i \right) $$

where $s$ is the kernel size (here, we set $s$ to 13), $A\left( n \right)$ represents the output of the Avg Pool at EEG sampling index $n$, which is the mean value of the EEG signal in the window from $n \,{\text{t}}o\,{ }n + s$. To ensure that the number of EEG data remained unchanged after average pooling, we used the repeat method to pad the input signal.

*3. Fully Connected Layer (FC)*

Following the Avg Pool layer, the output features were further processed and expanded into higher-dimensional features by FC-1, with an output dimension of 500. To enhance the model’s non-linear learning capability, we applied the ReLU non-linear activation function to the output of FC-1.

4 $$ F\left( n \right){ } = { }\mathop \sum \limits_{{i{ } = { }1}}^{N} W\left[ i \right] \times A\left[ i \right] + B\left[ i \right] $$

where $F$ represents the output of FC-1, $A$ is the output of Avg Pool, $W$ is the weight of FC-1, and $B$ is the bias coefficients of FC-1. The bias coefficients and weights of the fully connected layer were both obtained through training.

*4. Dropout Layer*

To prevent overfitting, we introduced a dropout layer where neurons were randomly dropped with a probability of $P{ } = { }0.9$. Dropout could also be used as a method to introduce noise to the model, which helps to enhance the model’s generalizability by preventing it from relying too heavily on specific local features.

*5. Output Layer*

This fully connected layer serves as the output layer with a dimension of 2, mapping the features to the complex domain and providing a complex expression of μ rhythm signals $Z$.

5 $$ Z\left( n \right){ } = { }\mathop \sum \limits_{{i{ } = { }1}}^{N} U\left[ i \right] \times C\left[ i \right] + D\left[ i \right] $$

$Z$ represents the output of the EPN, $C$ is the output of the dropout layer, $U$ is the weight of the output layer, and $D$ is the bias coefficients of the output layer. The bias coefficients and weights of the output layer were also learned through training.

### Dataset Generation and Procedure

#### Pre-recorded EEG Dataset

We studied existing data from twenty subjects (6 female) who received single-pulse TMS of the motor-hand area. Before the experiment, all the subjects provided written informed consent, which was approved by the National Institutes of Health Combined Neuroscience Section Institutional Review Board. The subjects were not intentionally preselected for μ rhythm spectral features in their EEG signals, as was done in another study [39, 40]. The electrophysiological signals and related navigation data are published in a database at https://openneuro.org/datasets/ds002094 created by Hussain *et al*. [29]. The scalp EEG signal was recorded at a 5 kHz sampling rate by a 32-channel TMS-compatible amplifier (BrainAmp MR+, Brain Vision, Gilching, Germany). At the beginning of the TMS, an additional 5-min resting-state EEG was recorded. This was used to train and test different phase prediction frameworks.

**Fig. 2:**
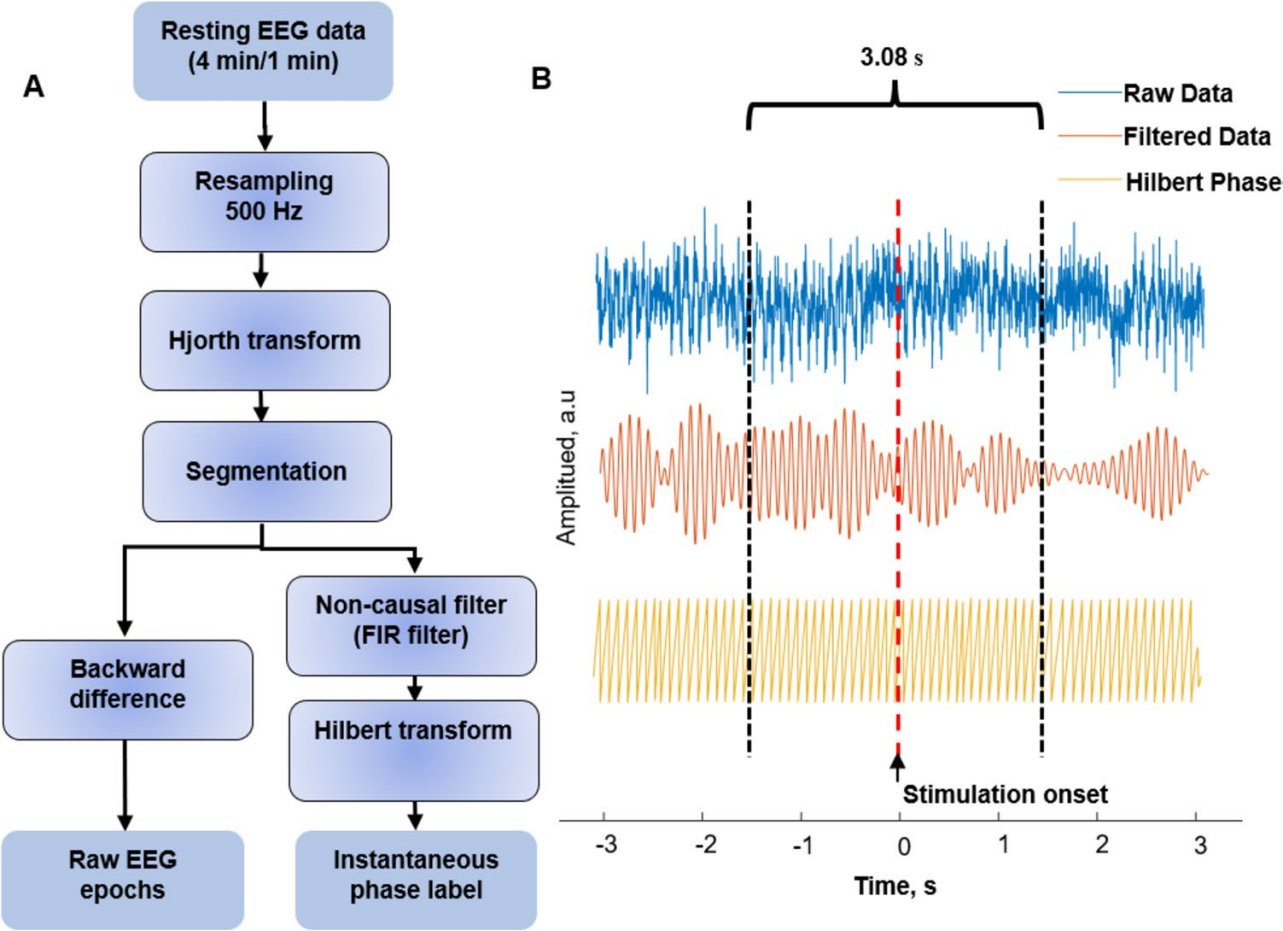
The generation of datasets and illustration of the instantaneous phase definition for the EPN, MLOF, and AR models. **A** The pipeline of generating training and testing datasets. The 5-min resting data is divided into a 4-min resting set for training and validation and a 1-min resting dataset. The details of dataset generation are described in the Dataset Generation and Procedure section. **B** Definition of the instantaneous phase label. The instantaneous phase (stimulation onset) is defined as the center of the EEG signal in the 6.16 s window after non-causal filtering and Hilbert transformation.

To generate the EPN, MLOF, and AR training datasets shown in Fig. 2A, we divided each subject’s 5-min resting data into a 4-min training and validation set and a 1-min test set and then resampled to 500 Hz followed by a 5-point C4-centered sum-difference Hjorth-Laplace transform as a spatial filter to capture the more local μ rhythm information [41]. The training and testing datasets were then separately segmented into windows 6.16 s long (3,080 sampling points) with a series of overlapping window lengths: 0 s (no overlap), 0.128 s (64 sampling points), 0.256 s (128 sampling points), 0.384 s (192 sampling points), 0.512 s (256 sampling points), 0.640 s (320 sampling points), 0.768 s (384 sampling points), 0.896 s (448 sampling points), 1.024 s (512 sampling points), 1.152 s (576 sampling points), and 1.280 s (640 sampling points). The ground truth of the μ rhythm was defined as the raw EEG data after applying a two-pass zero-phase finite impulse response (FIR) bandpass filter (8 Hz–13 Hz bandpass, 769 order). As Fig. 2B shows, 0 s is the center of a 6.16 s interval, which is treated as the onset event for the stimulation, the non-causally filtered data between −1.54 s and 1.54 s is not affected by the filter, and the Hilbert phase of the filtered data at time 0 s is defined as the phase ground truth. The instantaneous phase at the simulation onset at time 0 s was defined as the output label of the three models. The backward difference $y\left( n \right){ } = { }x\left( {n + 1} \right) - x\left( n \right)$ of the raw data from −0.5 s to 0 s, which was used to remove large electrode drifts and facilitate machine learning training speed [31], was defined as the inputs to the EPN and MLOF models, where $x\left( n \right)$ was the raw EEG data at index $n$. The raw data from −1.0 s to 0 s before stimulation onset was defined as the input to the AR model [27].

For the ETP model, each subject’s 5-min resting data was divided into a 4-min training and a 1-min test set and then resampled to 1000 Hz followed by a 5-point C4-centered sum-difference Hjorth-Laplace transform. The μ rhythm cycle of the frequency that is specific to each subject was determined by the first 4-min resting data, as detailed in [34]. Like the EPN, MLOF, and AR models, the next 1-min test set was also segmented by a window length of 3.08 s (3,080 sampling points) with a series of overlapping window lengths: 0 s (no overlap), 0.64 s (64 sampling points), 0.128 s (128 sampling points), 0.192 s (192 sampling points), 0.256 s (256 sampling points), 0.32 s (320 sampling points), 0.384 s (384 sampling points), 0.448 s (448 sampling points), 0.512 s (512 sampling points), 0.576 s (576 sampling points), and 0.64 s (640 sampling points). The filter order and ground truth definition were the same as in the EPN, MLOF, ETP, and AR models. The prediction phase of the ETP at time 0 s was interpolated using Equation (2).

Given that the EPN and MLOF models require appropriate training for phase prediction, we implemented and trained these models using the PyTorch framework. We applied the Adam optimizer with an initial learning rate of 10^−5^ to optimize the model’s hyperparameters. The mean squared error function was used as the loss function, and the models were trained for 3,000 epochs with a batch size of 512.

#### Simulated EEG Dataset

The ground truth phase of the μ rhythm is often defined as the Hilbert phase of non-causal filtering. However, because of EEG $1/f$ background neural noise, the non-causal phase estimate still has limitations with respect to obtaining the phase of the actual rhythm. To verify the performance of the EPN and other models with respect to the underlying real truth, we used the fitting oscillations & one over f (FOOOF) toolbox [42] to fit the background noise and the Kuramoto model [43] to model the EEG signal. The real truth phase was defined as the non-causally recovered phase after applying a Hilbert-transform, prior to the addition of noise. A detailed description can be found in [33].

### Real-time Phase Prediction Procedure

#### Human Subjects

Six healthy male subjects signed an informed consent document and participated in this study, which was approved by the Ethical Committee of the Institute of Automation, Chinese Academy of Sciences. They were asked to get a good rest and not to use any psychotropic substances or coffee before the experiment. Due to technical problems, one participant was not included in the subsequent analyses.

#### Experimental Protocol

The subjects were instructed to sit quietly with their eyes open during the recording. Initially, 5 min of resting EEG data was collected. We tested the performance of real-time μ rhythm phase prediction in four models (EPN, MLOF, AR, and ETP) at the rising edge (0°), peak (90°), falling edge (180°), and trough (270°) with 100 replications collected in each of the four sessions. The order of the algorithms in each session and the target phase selection were shuffled to obtain random order. There was a 5-min break after each session.

#### EEG Recording

EEG data were recorded with a 16-channel EEG amplifier (BRE-100, CASIA Brain, Beijing, China) and a 32-channel EEG cap (GT Cap PRO, Greentech, Wuhan, China). FC1, FC5, CP1, and CP5 electrodes around the C3 electrode were attached to the head (the FCz electrode was the reference and AFz was the ground electrode), and the impedance of each electrode was kept below 10 kΩ. The sampling rate was 1,000 Hz and the collected data were sent to a computer by USB cable.

#### Real-time Signal Processing and Model Deployment

The AR and ETP were implemented in custom MATLAB scripts (https://github.com/OpitzLab/CL-phase), and the EPN and MLOF models were trained by custom Python scripts and exported to an Open Neural Network Exchange file to share and deploy the models (https://github.com/onnx). The real-time EEG data were streamed to a processing computer (Z620, Hewlett-Packard, California, USA) by Lab Streaming Layer (https://github.com/sccn/labstreaminglayer). To reduce the computational latency of the EPN and MLOF machine learning models, we used Qt C++ (https://github.com/qt/qt5) and the CUDA Deep Neural Network library (https://github.com/NVIDIA/cudnn-frontend) to deploy these models into graphics processing unit (Geforce RTX 2060, Nvidia, California, USA) for real-time computation. Trigger signals were sent to the TMS machine (Super Rapid 2, Magstim, Carmarthenshire, UK) *via* the PCI parallel port (MCS9865, MosChip Semiconductor Technology, Telangana, India) which took only 1 ms–2 ms, and the latency was stable over time. We focused on the model performance of the phase prediction rather than on the output of the TMS stimulation, so the TMS machine was shut down and only the trigger time was labeled on the data stream. As a result, the real-time EEG data was not distorted, which helped us to build the non-causal toolchain and restore the ground truth of the μ rhythm [34].

### Evaluation

To obtain a comprehensive measure of each model’s performance, we applied the circular mean (MEAN), circular variance (VAR), standard circular deviation (SD), mean absolute circular error (MACE) [33, 44], and accuracy (ACC) [34]:

6 $$ {\text{MEAN}} = {\text{Arg}}\left( {\frac{1}{N}\mathop \sum \limits_{i = 1}^{N} {\text{e}}^{{i\theta_{{{\text{predict}}}} - i\theta_{{{\text{true}}}} }} } \right) $$

7 $$ {\text{VAR}} = 1 - \left| {\frac{1}{N}\mathop \sum \limits_{i = 1}^{N} {\text{e}}^{{i\theta_{{{\text{predict}}}} - i\theta_{{{\text{true}}}} }} } \right| $$

8 $$ {\text{SD}} = \sqrt { - 2 \times \log (1 - {\text{VAR}})} $$

9 $$ {\text{MACE}} = \frac{1}{N}\mathop \sum \limits_{i = 1}^{N} \left| {{\text{Arg}}\left( {{\text{e}}^{{i\theta_{{{\text{predict}}}} - i\theta_{{{\text{true}}}} }} } \right)} \right| $$

10 $$ {\text{ACC}} = 1 - \frac{1}{N \times 180}{\text{Arg}}^{ \circ } \left( {\left| {\mathop \sum \limits_{i = 1}^{N} {\text{e}}^{{i\theta_{{{\text{predict}}}} - i\theta_{{{\text{true}}}} }} } \right|} \right) $$

where *N* is the EEG block number, $\theta_{{{\text{predict}}}}$ is the prediction phase in radians, and $\theta_{{{\text{true}}}}$ is the true phase in radians. The closer the value of MEAN is to 0, the smaller the phase prediction deviation. The smaller the SD, the less the spread of the measured distribution. The closer the accuracy approaches 1, the more accurate the phase prediction becomes.

To compare the performance of the four models, we applied the Wilcoxon signed-rank test and the Bonferroni correction for multiple comparisons. We further compared the effect of the signal-to-noise ratio (SNR) on the prediction accuracy of the different models. We performed the following steps: (1) The power spectral density (PSD) was first estimated by Welch’s method over 1-s epoched data with a Hann window. (2) The SNR was calculated as the average PSD from 8 Hz to 13 Hz divided by the total PSD (2 Hz–45 Hz). (3) A linear regression model with accuracy was used as the dependent variable, and SNR was the independent variable.

### Data Availability

All relevant data and computation codes are available from the corresponding authors upon request.

## Results

We hypothesized that the EPN machine learning model incorporating conventional filtering as well as perceptron models would capture the distribution and nonlinear features of a subject’s rhythms and recover the phase information of the EEG directly from the original signals more accurately than the currently widely used MLOF, AR, and ETP models.

### Validation on Pre-recorded Data

**Fig. 3:**
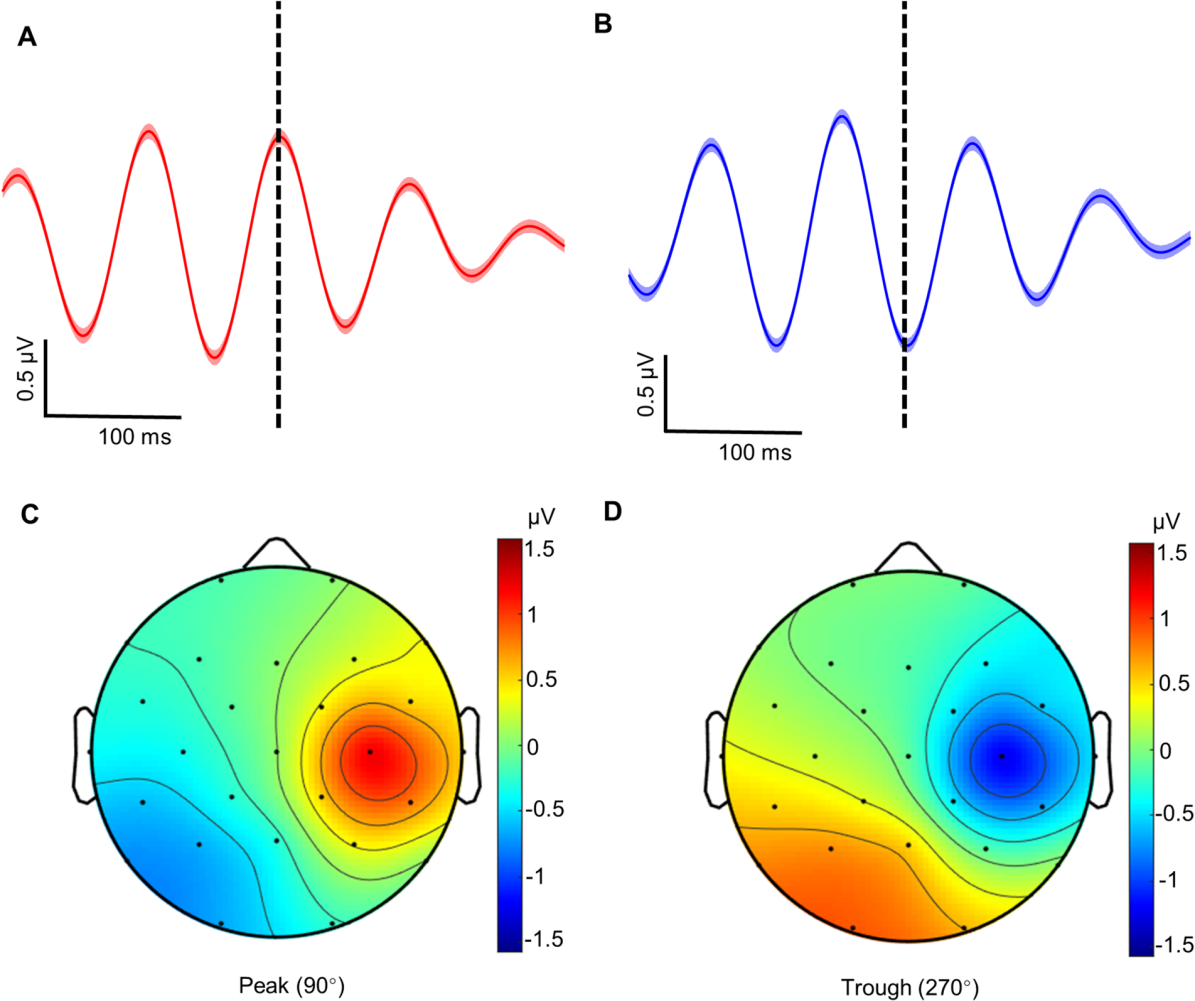
Phase categorization performance of the EPN model at the peak (90°) and trough (270°) on pre-recorded resting data. **A, B** Average peak and trough trials across 20 subjects. The vertical dashed line shows the center of the 6.16 s epoched data where the μ rhythm instantaneous phase is estimated. 0.4 of the 6.16 s is shown as shaded ± SEM. **C, D** Scalp distribution of the EEG activity during μ rhythm peaks and troughs calculated by the C4-Hjorth transformation with a non-causal bandpass filter. The topography shows the average μ rhythm signal activity centered around the predicted peaks and troughs using a ±10 ms window; amplitudes (in μV) are indicated on the color bar. Note that the peaks and troughs of the μ rhythm signal are localized over the right sensorimotor cortex.

We first determined whether the EPN model could categorize the μ rhythm into peaks (90°) and troughs (270°) as well as identify the EEG activity distribution features near the peaks and troughs. As shown in Fig. 3A and B, the EPN model successfully classified the trials into peaks and troughs, and the EEG activity during the μ peak and trough trials was also localized to the right central regions (Fig. 3C, D).

**Fig. 4:**
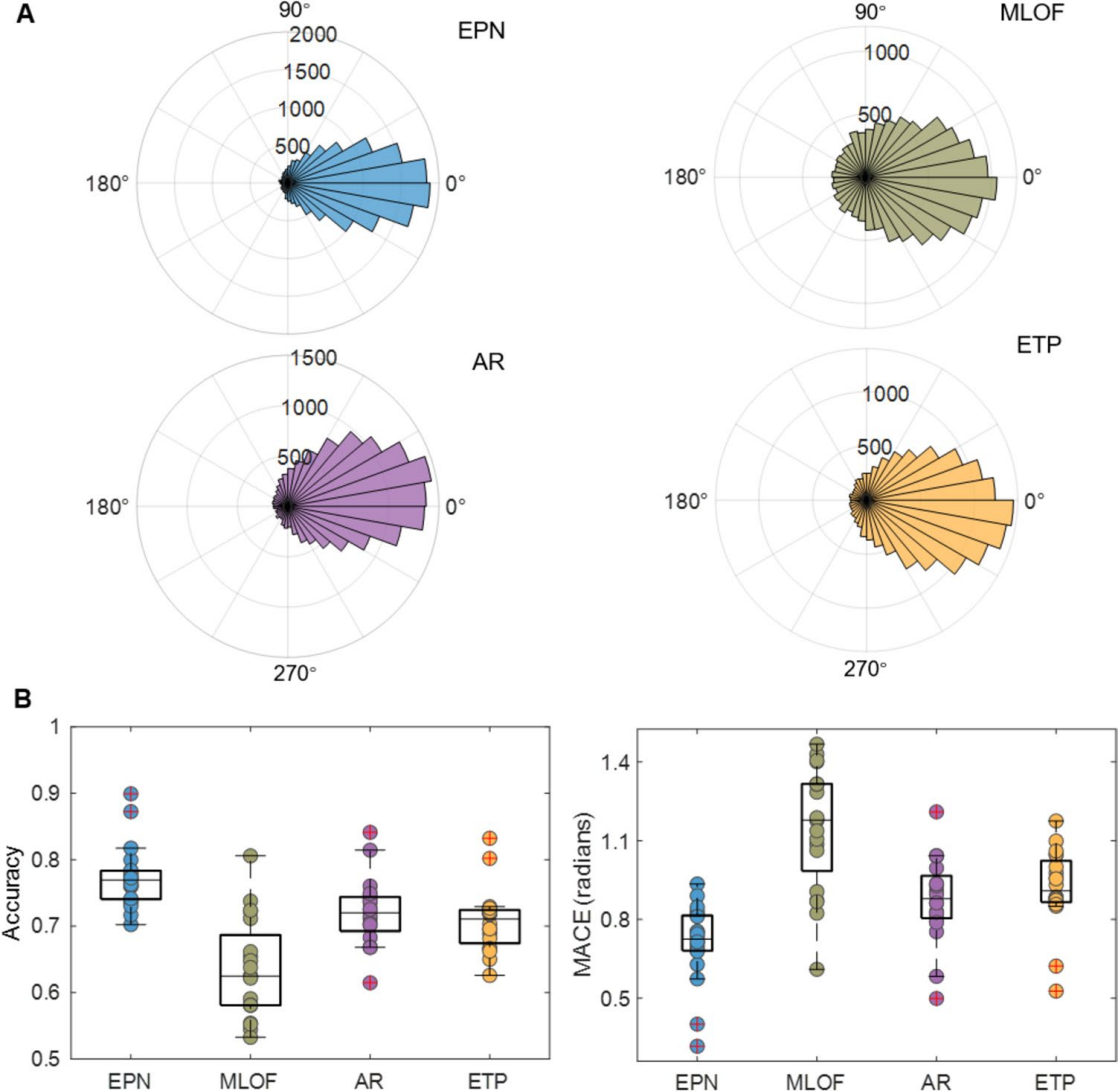
Phase prediction results in pre-recorded datasets. **A** Distribution of the difference between the prediction phase and the ground truth in the pre-recorded data. Qualitatively, the EPN model shows the least spread among the models. **B** Boxplot of the accuracy and MACE for the four models with the individual data points. The EEG phase prediction network (EPN) model has the highest accuracy and lowest mean absolute circular error (MACE) compared with the multi-layer filter architecture (MLOF), auto-regress (AR), and educated temporal prediction (ETP) models.

As Fig. 4A shows, the average difference between the prediction phase and the ground truth phase calculated by a non-causal filter and the Hilbert transform was close to zero, and EPN showed the least spread, implying that the EPN model had a smaller prediction variance. Quantitatively, the EPN model manifested the greatest accuracy and lowest MACE (mean accuracy = 77.34%, mean MACE = 0.71) compared with the MLOF (63.47%, 1.15), AR (72.08%, 0.88), and ETP (70.78%, 0.91) (Fig. 4B). Non-parametric pairwise tests with Bonferroni correction showed a statistically significant difference between both the accuracy and MACE of each algorithm pair (EPN *vs* MLOF: *P* <0.001, EPN *vs* AR: *P* <0.001, EPN *vs* ETP: *P* <0.001, MLOF *vs* AR: *P* <0.001, MLOF *vs* ETP: *P* = 0.002). In addition, the EPN model was able to complete the phase prediction within 2 ms at a 500 Hz sampling frequency, making it possible for the EPN to provide a real-time phase prediction. The performance test on the pre-recorded data is summarized in Table S1.

**Fig. 5:**
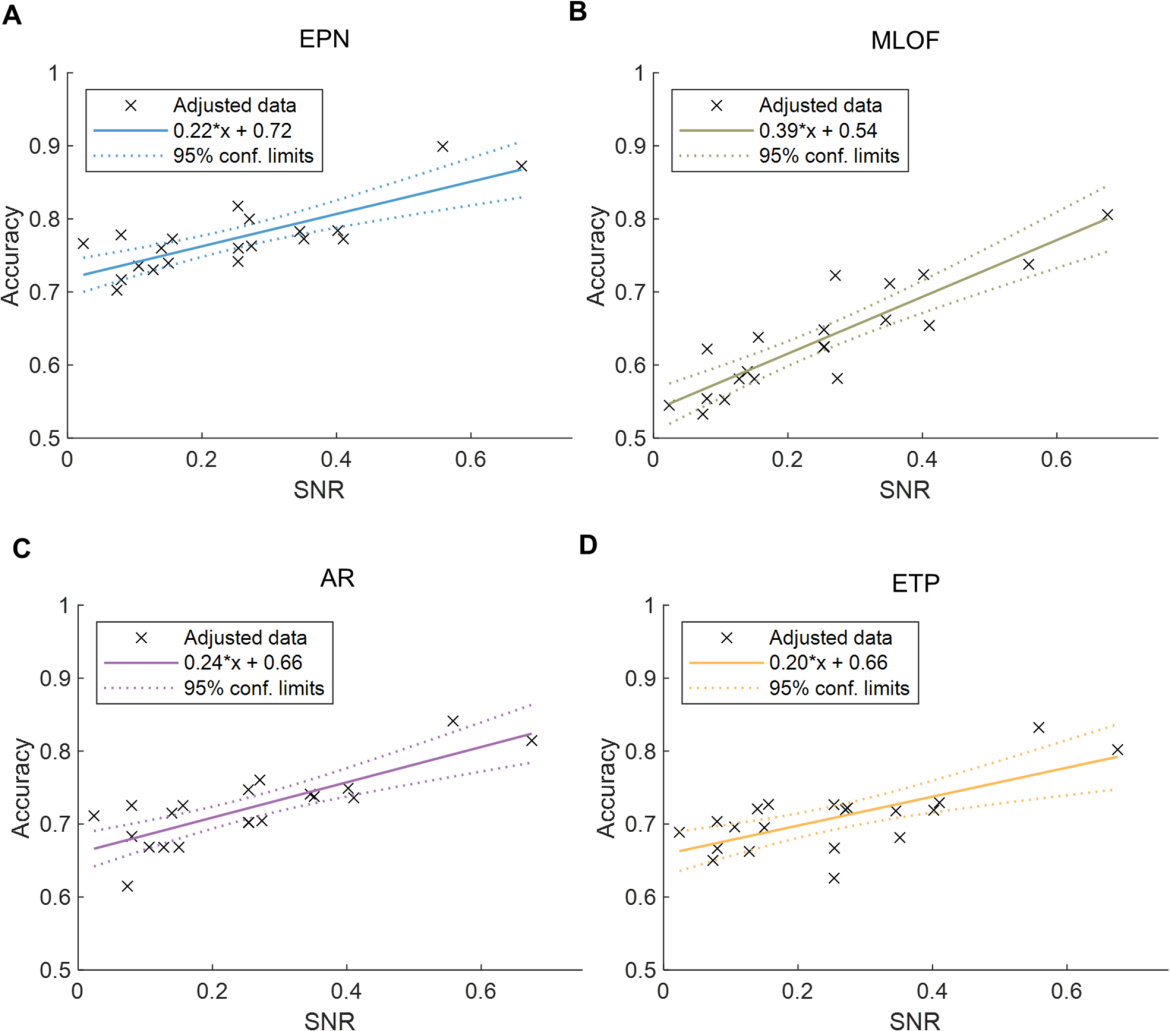
Linear regression results of the independent signal-to-noise ratio (SNR) and response accuracy of the phase prediction for each model. The linear regression is shown by colored solid lines and the 95% confidence limits are shown by the dashed lines. When the SNR increases, the accuracy of all the models improves significantly. **A,** EEG phase prediction network (EPN); **B**, multi-layer filter architecture (MLOF); **C**, auto-regress (AR); **D**, educated temporal prediction (ETP).

All four models had a significant positive relationship between the SNR and accuracy (EPN: *R*^2^_adj_ = 0.61, *P* <0.001; MLOF: *R*^2^_adj_ = 0.78, *P* <0.001; AR: *R*^2^_adj_ = 0.64, *P* <0.001; ETP: *R*^2^_adj_ = 0.47, *P* <0.001) (Fig. 5). This is similar to previous research [33, 34]. The prediction accuracy and coefficients for the four models are as follows: EPN (77.34%, 0.22), ETP (63.47%, 0.39), AR (72.08%, 0.24), and MLOF (70.78%, 0.20). These indicators also suggest that the EPN model maintains a higher prediction accuracy and exhibits lower sensitivity to variations in rhythm strength than both the MLOF and AR models.

We also established EPN models with 17 distinct parameter configurations, as listed in Table S2. These models were retrained, and the resulting loss curves for two randomly-selected participants are illustrated in Fig. S1. It is evident that as training epochs increase, the training loss decreases consistently across all parameter configurations of the EPN model, indicating that the models were appropriately trained. Moreover, we assessed the performance of all models on an independently partitioned test set (Table S3) and found that the circular mean across all EPN models was about −1°, with an SD of ~54° and a MACE of ~0.72, resulting in an accuracy of ~77%. Among these models, EPN-1, which corresponds to the parameters reported in the main text, yielded reproducible training results (circular mean −0.98°, SD 53.44°, MACE 0.71, accuracy 77.29%) very close to those reported in the main text (−0.94°, 53.34°, 0.71, 77.34%). The result also suggested that the EPN model is not sensitive to parameter selection. In addition, EPN-1, the model chosen in our main text, has been appropriately trained, affirming the correctness of the parameter selection.

### Validation on Simulated Data

**Fig. 6:**
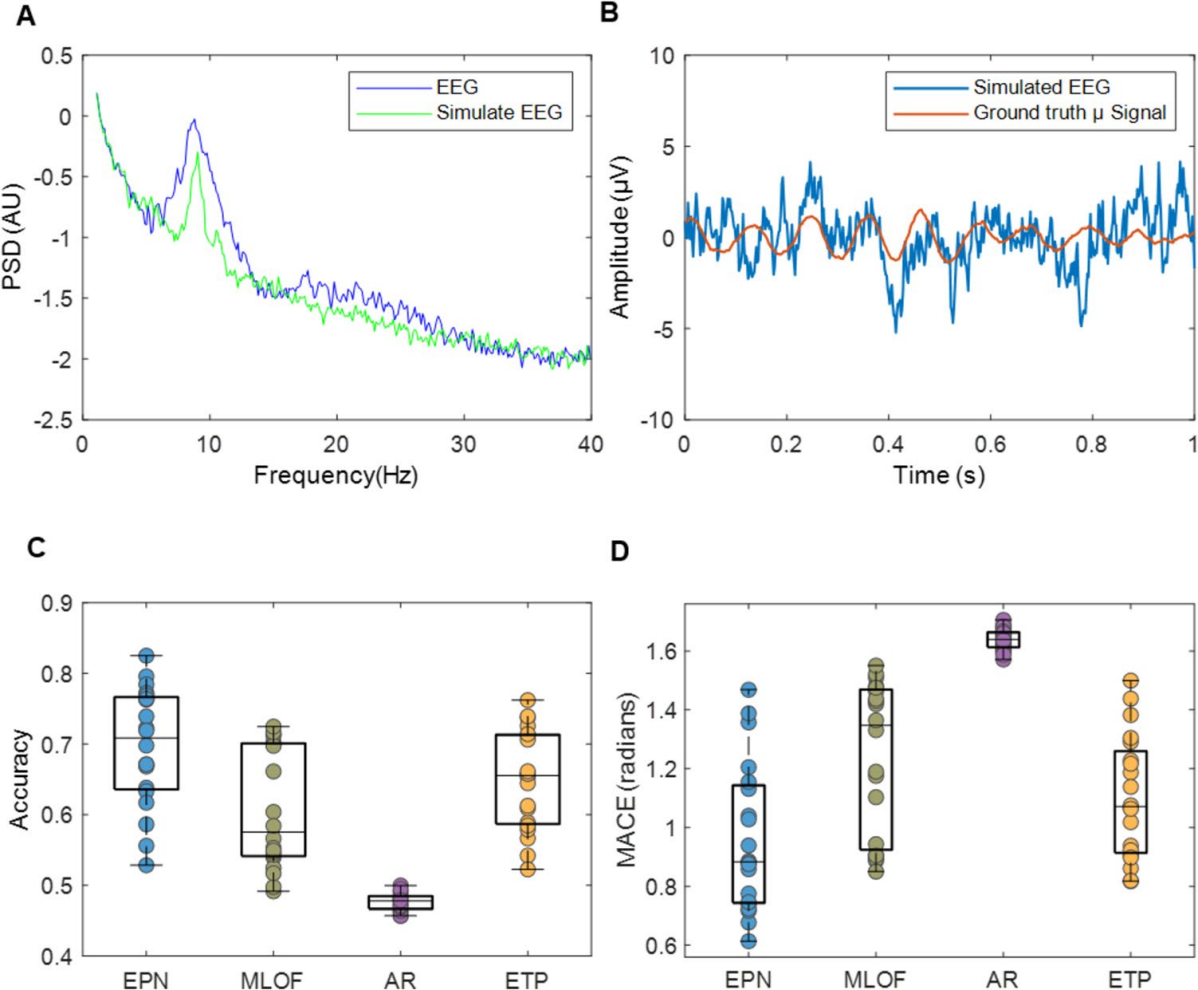
EEG modeling and prediction results on simulated data. **A** The power spectral density (PSD) for a subject from the original EEG data and the corresponding PSD of the simulated EEG. **B** Simulated time series EEG data with the underlying ground-truth signal. **C**, **D** Boxplots of the accuracy and mean absolute circular error (MACE) for the four models using individual data points. The EPN model manifests the greatest accuracy and the lowest MACE among the four models on simulated data. EPN, EEG phase prediction network; MLOF, multi-layer filter architecture; AR, auto-regress; ETP, educated temporal prediction.

Because of $1/f$ background neural noise in the EEG signal, the Hilbert phase calculated from the non-causal bandpass-filtered signal may fail to reflect the true phase information. We therefore reconstructed the noise and the real truth μ rhythm data (Fig. 6A, B) on a pre-collected dataset and examined the prediction performance of the different models on the simulated dataset. The EPN, MLOF, and ETP models were trained on the Hibert phase of the real truth signal and showed a smaller spread (Fig. S2), greater accuracy (Fig. 6C), and a lower MACE (Fig. 6D) than the AR models, which lacked feedback from the true phase in the training session. The EPN model also manifested the greatest accuracy and lowest MACE (mean accuracy = 69.43%, mean MACE = 0.96) compared with the MLOF (60.47%, 1.24), AR (47.80%, 1.64), and ETP (63.24%, 1.13) on simulated data. Non-parametric pairwise tests with Bonferroni correction showed a statistically significant difference between both the accuracy and MACE of each algorithm pair (EPN *vs* MLOF: *P* <0.001, EPN *vs* AR: *P* <0.001, EPN *vs* ETP: *P* <0.001, MLOF *vs* AR: *P* <0.001, MLOF *vs* ETP: *P* = 0.002, AR *vs* ETP: *P* <0.001). A detailed comparison of the results for all the models is presented in Table S4. Meanwhile, much like the pre-recorded dataset, the real-time dataset showed a positive relationship between SNR and accuracy in the EPN, MLOF, and ETP models (EPN: *R*^2^_adj_ = 0.77, *P* <0.001; MLOF: *R*^2^_adj_ = 0.53, *P* <0.001; ETP: *R*^2^_adj_ = 0.75, *P* <0.001), but this was not true in the AR model which showed a negative relationship (AR: *R*^2^_adj_ = 0.18, *P* = 0.035), as shown in Fig. S3.

### Validation on Real-time Experiment Data

**Fig. 7:**
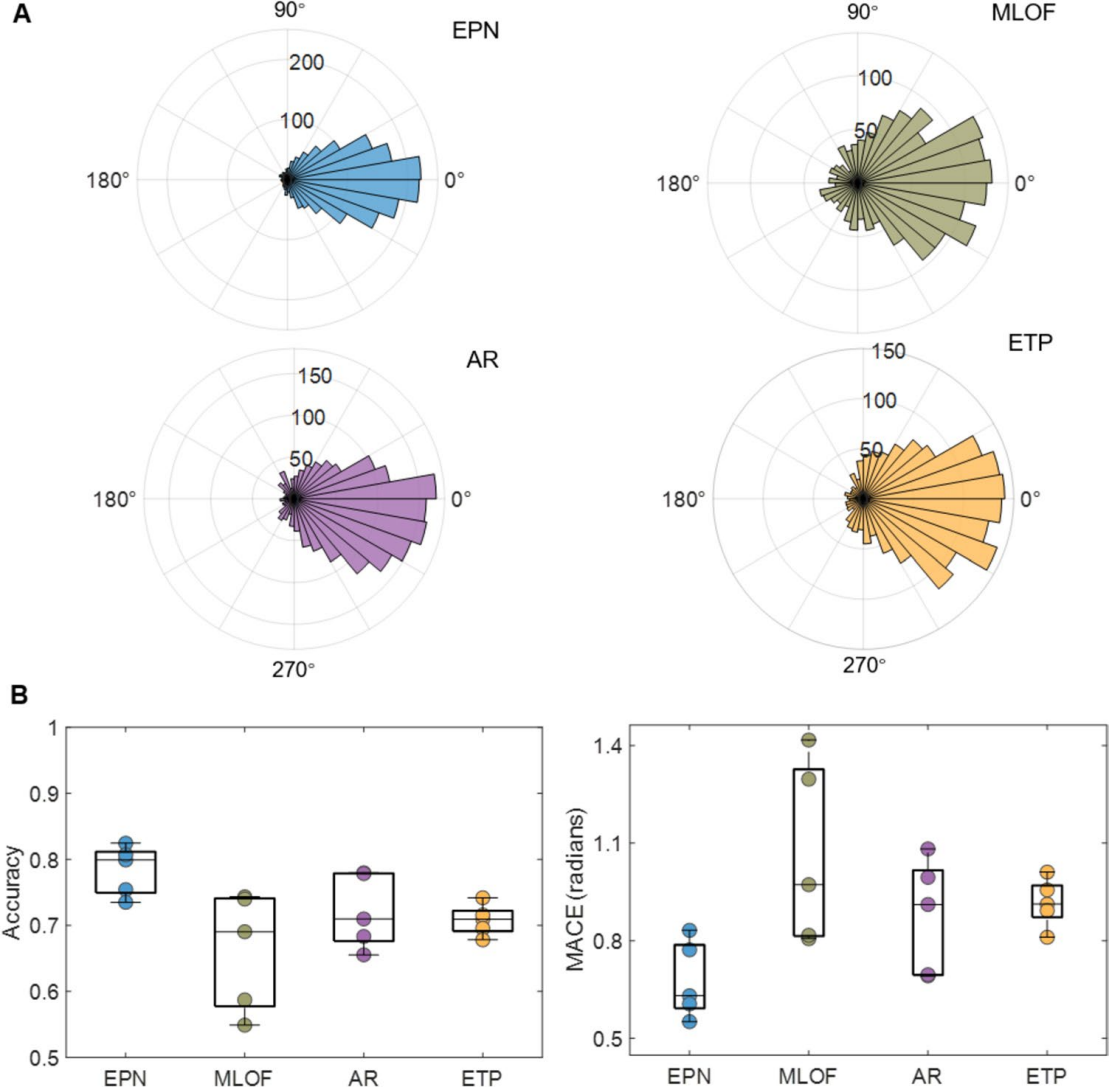
Phase prediction results for the real-time dataset. **A** Distribution of the difference between the prediction phase and the ground truth in the pre-recorded data. Qualitatively, the EPN model shows the least spread among the models. **B** Boxplots of the accuracy and mean absolute circular error (MACE)for the four models with the individual data points. The EEG phase prediction network (EPN) model has the highest accuracy and lowest MACE compared with the multi-layer filter architecture (MLOF), auto-regress (AR), and educated temporal prediction (ETP) models.

We developed a novel method of machine learning deployment and validated the performance of the four models in a real-time EEG signal acquisition environment. As shown in Fig. 7A, the mean difference between the prediction phase and ground truth phase was closer to zero for the EPN than for the other models. The EPN model achieved the greatest accuracy and lowest MACE (mean accuracy = 78.40%, mean MACE = 0.68) compared with the MLOF (66.20%, 1.06), AR (72.14%, 0.88), and ETP (70.82%, 0.92) (Fig. 7B). The EPN also manifested the least spread in specific phases [rising edge (0°), peak (90°), falling edge (180°), and trough (270°)] (Fig. S4). In addition, non-parametric pairwise tests with Bonferroni correction showed a statistically significant difference between both the accuracy and MACE of each algorithm pair (EPN *vs* MLOF: *P* <0.001, EPN *vs* AR: *P* <0.001, EPN *vs* ETP: *P* <0.001, MLOF *vs* AR: *P* = 0.035). The prediction results for the rising edge, the peak, the falling edge, and the trough are summarized in considerable detail in Table S5. Similar to the pre-recorded EEG dataset, the EPN, MLOF, and AR models, but not the ETP, also exhibited a significantly positive relationship between the SNR and accuracy (EPN: *R*^2^_adj_ = 0.54, *P* <0.001; MLOF: *R*^2^_adj_ = 0.53, *P* <0.001; AR: *R*^2^_adj_ = 0.70, *P* <0.001; ETP: *R*^2^_adj_ = −0.03, *P* = 0.54) (Fig. S5).

## Discussion

Adjusting external stimuli or neuromodulatory devices according to the instantaneous phase of an EEG rhythm is a very promising field. However, the accuracy of existing phase predictions requires improvement, particularly in non-preselected subjects and trials. In this paper, we present a novel phase prediction model called EPN that can capture the dynamic feature of EEG rhythms and directly map the instantaneous phase from the narrow-band EEG data. We examined the performance of the four models on three datasets. On the pre-recorded and simulated EEG dataset, the EPN achieved the lowest MACE and the greatest accuracy of the four models. The processing delay was within 1 sampling interval (500 Hz sampling rate), satisfying the real-time requirement. Then, we developed a method of machine learning deployment on a desktop computer and validated the performance of the four models in a real-time EEG signal-acquisition environment. As with the pre-recorded and simulated EEG datasets, the EPN manifested the greatest accuracy in both the average and specific phases (phases 0°, 90°, 180°, and 270°) of the four models. We believe that the EPN model outperforms the other three methods (MLOF, AR, ETP) primarily due to its architecture. The second layer of the EPN model consists of an average pooling layer, which functions similarly to a moving average filter, suppressing noise and enhancing the model’s robustness [45]. In addition, to enhance the model’s non-linear learning capability, the ReLU non-linear activation function was applied to the output of the fully connected layer [46]. More importantly, unlike traditional models like AR, EPN was retrained for each subject, thus generating subject-specific and personalized phase prediction models. These robust results indicated that the EPN learned the non-stationary EEG features of the whole subject population rather than utilizing only a small window of signal features.

As the order of the AR model is constant over a fixed time window, the dynamic EEG features across the time windows and individuals cannot be learned [27]. In addition, the real-time computation limits the optimization of the parameter dynamics. Also, the order of the non-causal two-pass filter used in the AR model should not be set too large, or it would significantly delay the computation, affecting the phase prediction performance in a real-time environment.

We were unable to reproduce the finding that the accuracy of the ETP prediction was slightly greater than that of AR on the pre-recorded data and the real-time experiment [34]. This may have been because the educated phase bias learned in the resting session had little effect on improving the prediction accuracy of the stimulation trials in our dataset. ETP uses a zero-phase shift filter (time domain filter) and a brick-wall filter (frequency domain filter) separately to denoise 3-min resting EEG data in the learning phase. These two types of phases calculated by the Hilbert transform were defined as the ground truth phase and the biased phase. To reduce the systematic phase error called phase bias between the two methods at the distorted edge of the data, the ETP used a full cycle search method to reduce the mean absolute error. Then, the instantaneous phase was linearly interpolated using the undistorted peak phase plus the phase bias. Although the peak-finding computed phase of the ETP and the inherent bias of the filter were gapped by the pre-acquisition data, the non-stationary and transient nature of the EEG signals was likely to have led to a failure to apply the learning from the pre-acquisition session to the used real-time computation phase; this may have affected the accuracy of the ETP.

The MLOF was also introduced to capture the causal mapping from a raw EEG recording to a phase estimate generated with a non-causal signal processing chain [33]. MLOF was initially devised to predict the alpha-rhythm phase in the occipital region in offline analyses. The key feature of this method is to combine two FC layers, one of which uses a Softmax operation, *via* a dot-multiplying operation to detect multiple frequency representations. In this study, we used a novel machine learning application to deploy this method and made the application available to predict the μ rhythm phase of the sensorimotor area in a real-time environment. MLOF uses the IAF of individual subjects by suppressing irrelevant frequencies. However, since the alpha rhythm in the occipital area usually manifests a lower peak frequency and less asymmetry and has a higher amplitude than the μ rhythm in the sensorimotor area, the μ rhythm phase prediction of the MLOF model in the sensorimotor region may be poorer than expected [47–49].

Closed-loop neuromodulation techniques such as TMS can causally assess the functions of the oscillatory brain rhythms in perceptual and cognitive processes [50, 51]. Triggering TMS at a specific phase of the instantaneous alpha oscillation of the left dorsolateral prefrontal cortex is feasible and safe in the treatment of major depressive disorder [52], and shows increased entrainment over sessions for the anterior cingulate [26]. Currently, the low accuracy of current phase prediction methods may limit the usefulness of joint EEG and TMS studies. Meanwhile, as has previously been reported [32, 34], we also found that the SNR affects the predictive effectiveness of the model, e.g., the prediction accuracies of the four models on the pre-recorded dataset were all significantly positively correlated with the SNR. This led to some studies having to screen out individuals with a relatively high SNR in order to make the model’s predictions sufficiently accurate. However, this pre-screening operation has limited the use of TMS and closed-loop EEG modulation in the general population and even led to irreconcilable and conflicting conclusions. For example, the phase of the μ rhythm shaping the excitability of the sensorimotor cortex states is still contradictory. The TMS-evoked MEP amplitude was significantly greater at the trough of the μ rhythm than at the peak in the sensorimotor area [27, 28]. However, some research teams have not been able to reproduce these results and found complicated interactions between the sensorimotor oscillatory phase and power [29–31, 53]. In short, compared with the current, widely used methods (AR, ETP, and MLOF), EPN achieves the greatest accuracy in non-preselected subjects and trials, which we hope may throw light on these types of EEG phase-gating TMS studies. This may further advance the research on the relationship between the MEP and the EEG phase.

The ability of the EPN model to predict the EEG rhythm phase within the 8 Hz–13 Hz range in real-time suggests its potential applicability beyond the μ rhythm in the motor cortex to capture a broader range of alpha rhythm signals. Alpha oscillation (8 Hz–12 Hz) is commonly recorded in the posterior regions of the brain, particularly in the visual cortex, and is closely associated with various cognitive functions [54–57]. Some studies have also indicated that the phase of the alpha rhythm is correlated with stimulus presentation and information processing such as in working memory tasks [58–60]. Utilizing the phase information provided by the EPN model, we can precisely control the timing of TMS stimulation, facilitating the causal manipulation of brain activity and enabling further exploration of the relationship between real-time EEG phase and cognitive processes.

Moreover, EEG phase-triggered closed-loop neuromodulation methods based on the EPN model not only aid in understanding the neural mechanisms of cognitive functions but may also contribute to therapy for related disorders such as ADHD (attention deficit hyperactivity disorder). The phase of the alpha rhythm is associated with the execution of attention tasks, particularly in maintaining attention and suppressing external interference [61, 62]. In ADHD patients, dysregulation of the alpha rhythm during task performance may contribute to attention deficits and difficulties in attention control [63–66]. Utilizing the alpha phase prediction of the EPN model for TMS modulation in ADHD patients may offer valuable therapeutic benefits in ameliorating attention deficits. Thus, the EPN model demonstrates considerable clinical and research potential in the field of closed-loop modulation in TMS-EEG co-registration studies. Subsequent behavioral experiments will further extend the scope of applications of this model.

Our method also has the disadvantage that a 5-min period of resting EEG data has to be pre-collected to train the model. But this process is worthwhile. Since the number of model parameters for the EPN is not very large, the training of the model on an average graphics card (such as NVIDIA GeForce RTX 2060) takes only a few minutes. It is worth noting that other methods are available that can be used to calculate the instantaneous phase, such as the fast Fourier transform [29, 34] and the continuous wavelet transform [67] or even the use of a customized Digital Signal Processor chip to achieve phase prediction with ultra-low power consumption [68]. However, a review has shown that these methods may fail to capture the non-linear EEG features and may not be better than ETP or other machine learning-based models [34].

In summary, we focused on EEG phase prediction and proposed a machine learning model, which we called the EPN, to directly predict the instantaneous phase from raw EEG data. The EPN model manifests reliable prediction and validity with great accuracy. Due to the parallel nature of graphics card computations, the time required to compute one channel and multiple channels is about the same, so in the future, we will try phase prediction for multiple brain regions.

## Supplementary Information

Below is the link to the electronic supplementary material.

Supplementary file1 (PDF 1266 kb)
