# Supplementary material for: A Novel Real-time Phase Prediction Network in EEG Rhythm
Source: Neurosci Bull. 2024 Nov 29;41(3):391–405. doi: 10.1007/s12264-024-01321-z (PMC11876478; doi:10.1007/s12264-024-01321-z)
Supplement: Supplementary file 1 — Supplementary file1 (PDF 1266 kb) [file 12264_2024_1321_MOESM1_ESM.pdf]

Supplementary Materials

Supplementary Tables

Table S1 Prediction results and computation time of different models on pre-recorded data epochs

	Mean°	SD°	MACE (radians)	Accuracy	Time (ms)
**EPN**	**−0.94**	**53.34**	**0.71**	**77.34%**	**0.51**
MLOF	−2.94	85.66	1.15	63.47%	0.49
AR	−11.74	63.11	0.88	72.08%	1.12
ETP	−8.17	66.62	0.91	70.78%	0.23

EPN, EEG phase prediction network; MLOF, multi-layer filter architecture; AR, auto-regress; ETP, educated temporal prediction.

Table S2 EPN models with different parameters

	Input length	Avg pool	FC-1	Dropout rate
**EPN-1**	**250**	**13**	**500**	**0.9**
EPN-2	125	13	500	0.9
EPN-3	500	13	500	0.9
EPN-4	250	25	500	0.9
EPN-5	250	7	500	0.9
EPN-6	250	13	500	0.6
EPN-7	250	13	500	0.3
EPN-8	250	13	250	0.9
EPN-9	250	13	125	0.9
EPN-10	250	13	250	0.6
EPN-11	250	13	250	0.5
EPN-12	250	13	250	0.4

EPN-13	250	13	250	0.3
EPN-14	125	13	250	0.2
EPN-15	125	13	250	0.9
EPN-16	125	13	250	0.6
EPN-17	125	13	250	0.3

EPN-1 is the model parameter used in the main text, while EPN-2 to EPN-17 are the models with the same architecture as EPN-1 but with different parameters. Taking the EPN-1 model as an example, the input length is 250 samples, the kernel size of average pooling is 13, the output size of FC-1 is 500, and the dropout rate is 0.9. EPN, EEG phase prediction network.

Table S3 The prediction performance of EPN models with 17 different parameters

	Mean°	SD°	MACE (radians)	Accuracy
**EPN-1**	**−0.98**	**53.44**	**0.71**	**77.28%**
EPN-2	−0.92	54.28	0.73	76.87%
EPN-3	−0.98	54.27	0.73	76.87%
EPN-4	−0.67	53.34	0.71	77.33%
EPN-5	−1.10	54.15	0.71	76.96%
EPN-6	−0.90	53.46	0.71	77.28%
EPN-7	−0.96	53.41	0.71	77.30%
EPN-8	−1.06	53.56	0.72	77.22%
EPN-9	−1.10	53.68	0.72	77.14%
EPN-10	−1.01	53.79	0.72	77.13%
EPN-11	−0.96	54.19	0.72	76.96%
EPN-12	−0.95	54.51	0.73	76.82%
EPN-13	−0.87	54.81	0.73	76.69%
EPN-14	−0.99	55.23	0.74	76.49%
EPN-15	−0.97	54.47	0.73	76.80%

EPN-16	−0.75	54.58	0.73	76.75%
EPN-17	−0.64	54.91	0.73	76.62%

EPN, EEG phase prediction network.

Table S4 The prediction performance of different models on simulated data epochs

	Mean°	SD°	MACE (radians)	Accuracy
**EPN**	**−0.41**	**70.64**	**0.96**	**69.43%**
MLOF	1.15	94.69	1.24	60.47%
AR	141.0	132.09	1.64	47.80%
ETP	−3.20	81.44	1.13	63.24%

EPN, EEG phase prediction network; MLOF, multi-layer filter architecture; AR, auto-regress; ETP, educated temporal prediction.

Table S5. The prediction performance of different models in a real-time experiment.

	Target phase	Mean°	SD°	MACE (radians)	Accuracy
**EPN**	0°	−2.26	52.08	0.67	77.78%
	90°	−0.13	48.50	0.64	79.50%
	180°	−0.38	52.85	0.70	77.65%
	270°	−4.41	49.32	0.67	78.68%
	**All**	**1.62**	**50.73**	**0.68**	**78.40%**
MLOF	0°	−4.44	74.92	1.03	67.17%
	90°	−0.50	75.95	1.04	66.87%
	180°	7.38	84.34	1.14	63.78%

	270°	1.32	76.65	1.04	67.00%
	All	−0.59	78.00	1.06	66.20%
AR	0°	0.77	61.51	0.82	73.78%
	90°	−18.02	57.33	0.83	73.67%
	180°	−0.43	68.62	0.93	70.32%
	270°	24.64	61.97	0.92	70.79%
	All	−11.04	63.29	0.88	72.14%
ETP	0°	−3.52	64.59	0.89	71.32%
	90°	−4.42	68.69	0.95	69.83%
	180°	−5.05	65.05	0.89	71.56%
	270°	−5.00	68.38	0.93	70.27%
	All	−4.49	66.67	0.92	70.82%

EPN, EEG phase prediction network; MLOF, multi-layer filter architecture; AR, auto-regress; ETP, educated temporal prediction.

Supplementary Figures

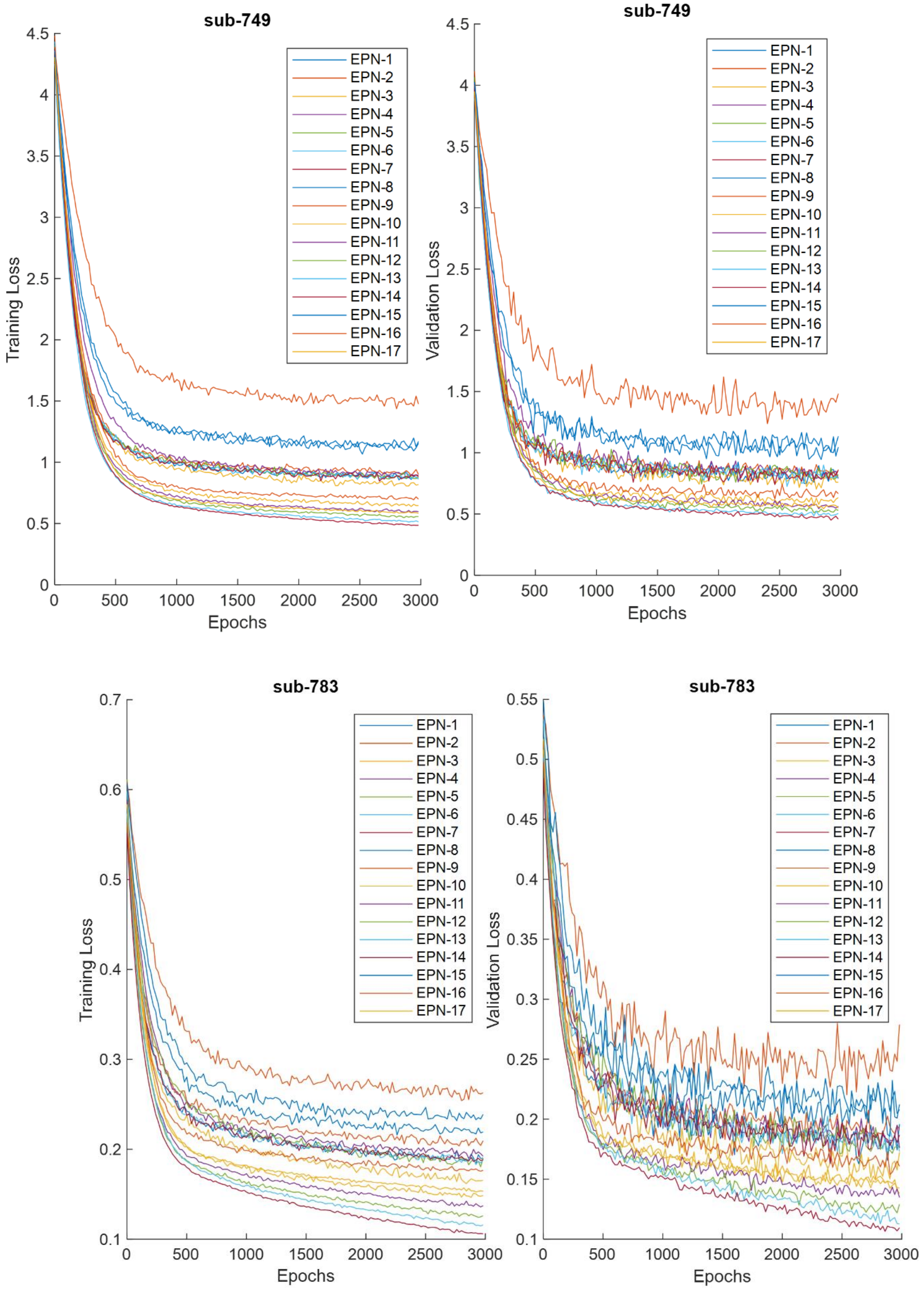

Fig. S1 The training and validation loss of EPN models on Subject 749 and Subject 783. As the training epochs increase, the training loss for all parameter combinations of the EPN model decrease, indicating that the models are appropriately trained. EPN, EEG phase prediction network.

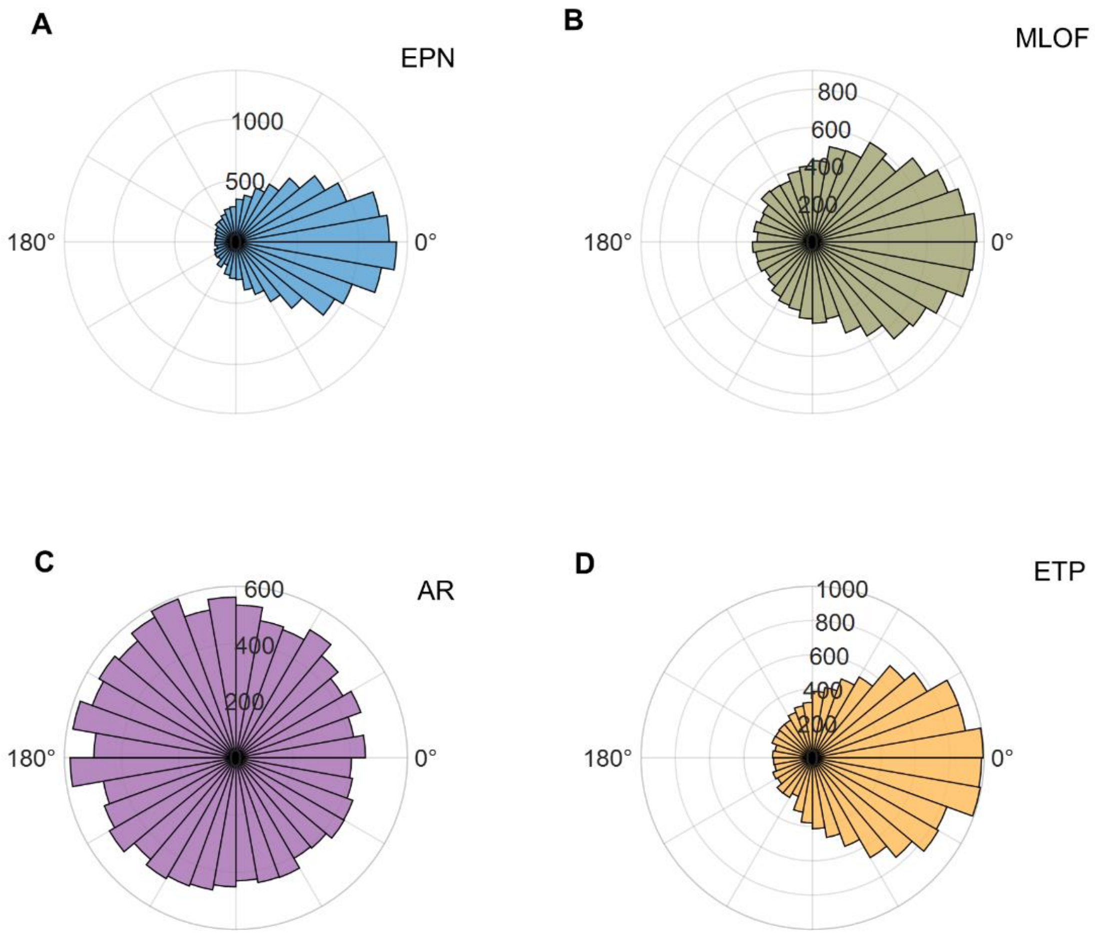

Fig. S2 Distribution of the difference between the predicted phase and the ground truth phase on simulated data. The narrower the spread from the distribution of the phase estimation to zero, the more accurate the model prediction. Qualitatively, the EPN (**A**) manifested the least spread compared with MLOF (**B**), AR (**C**), and ETP (**D**). EPN, EEG phase prediction network; MLOF, multi-layer filter architecture; AR, auto-regress; ETP, educated temporal prediction.

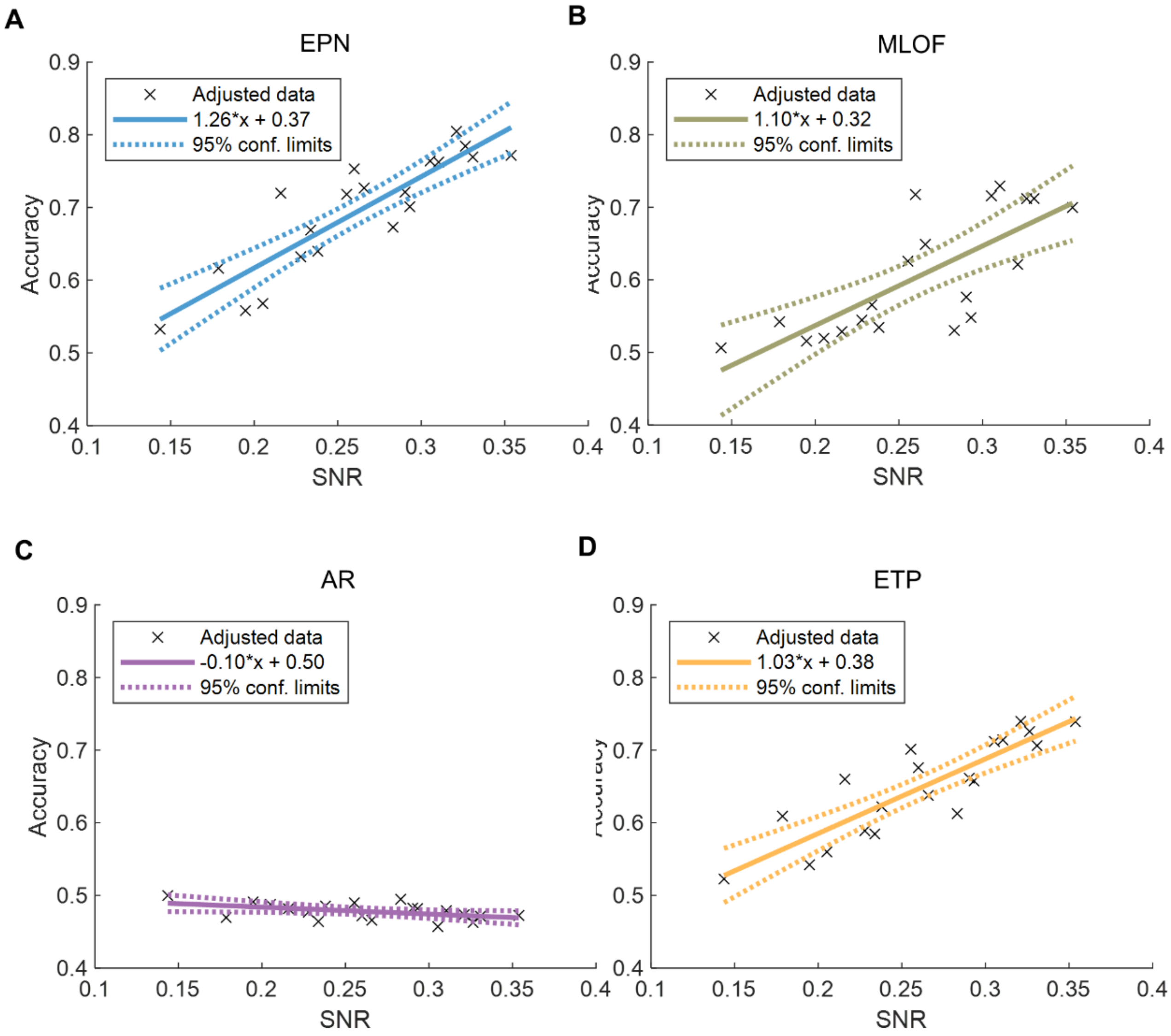

Fig. S3 Dependence between the phase estimation accuracy and the SNR in simulated data. There is a significantly positive relationship between the SNR and the phase estimation accuracy of the EPN (**A**), MLOF (**B**), and ETP (**C**) models, but the AR (**D**) model shows a negative relationship. EPN, EEG phase prediction network; MLOF, multi-layer filter architecture; AR, auto-regress; ETP, educated temporal prediction.

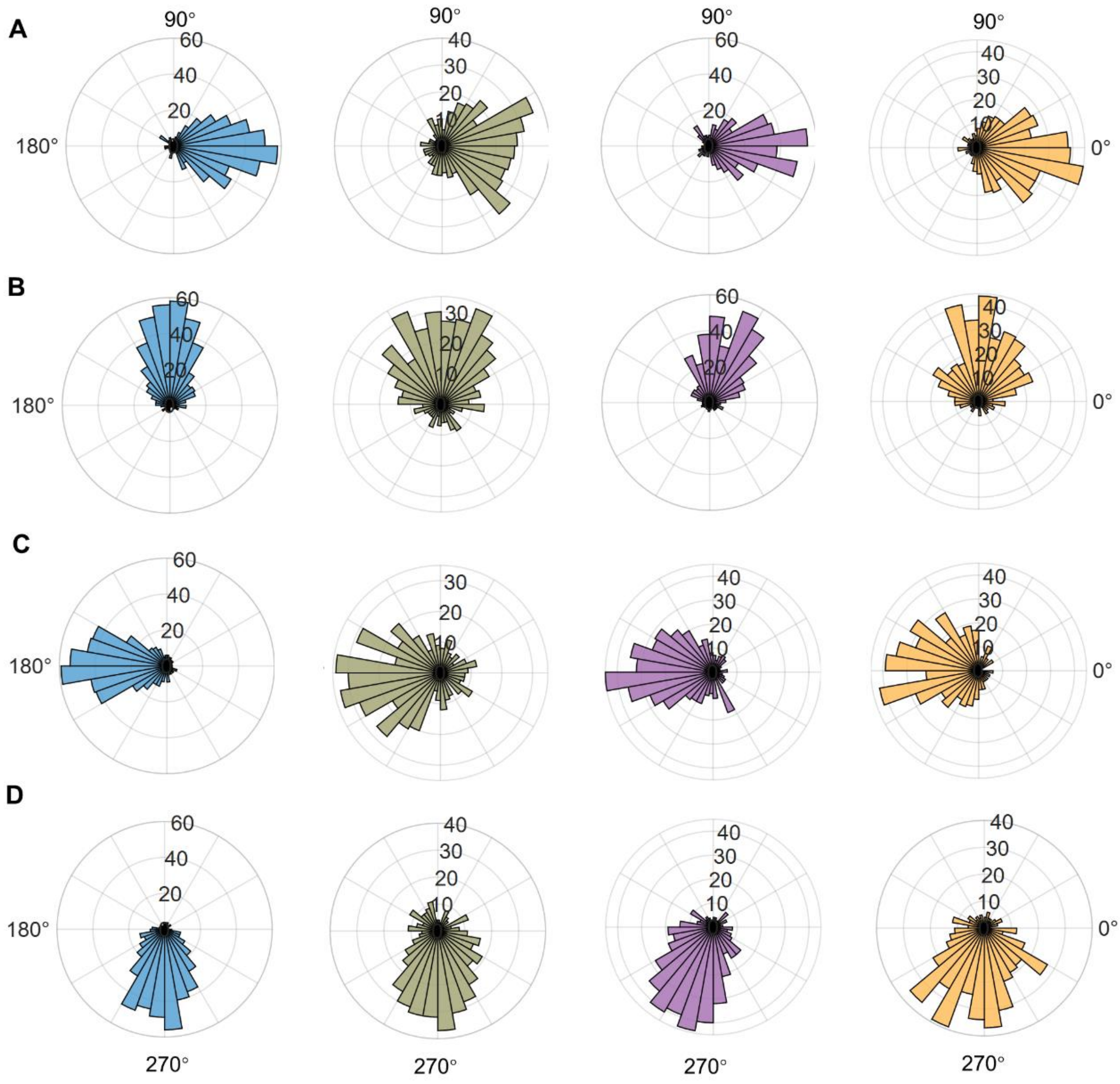

Fig. S4 Distribution of the difference between the predicted phase and the ground truth phase in a real-time experiment at specific phases (0°, 90°, 180°, and 270°). Qualitatively, the EPN manifests the least spread, lowest MACE, and greatest accuracy compared with the other three models when targeted at the rising edge 0° (**A**), peak 90° (**B**), falling edge 180° (**C**), and trough 270° (**D**) phases. EPN, EEG phase prediction network.

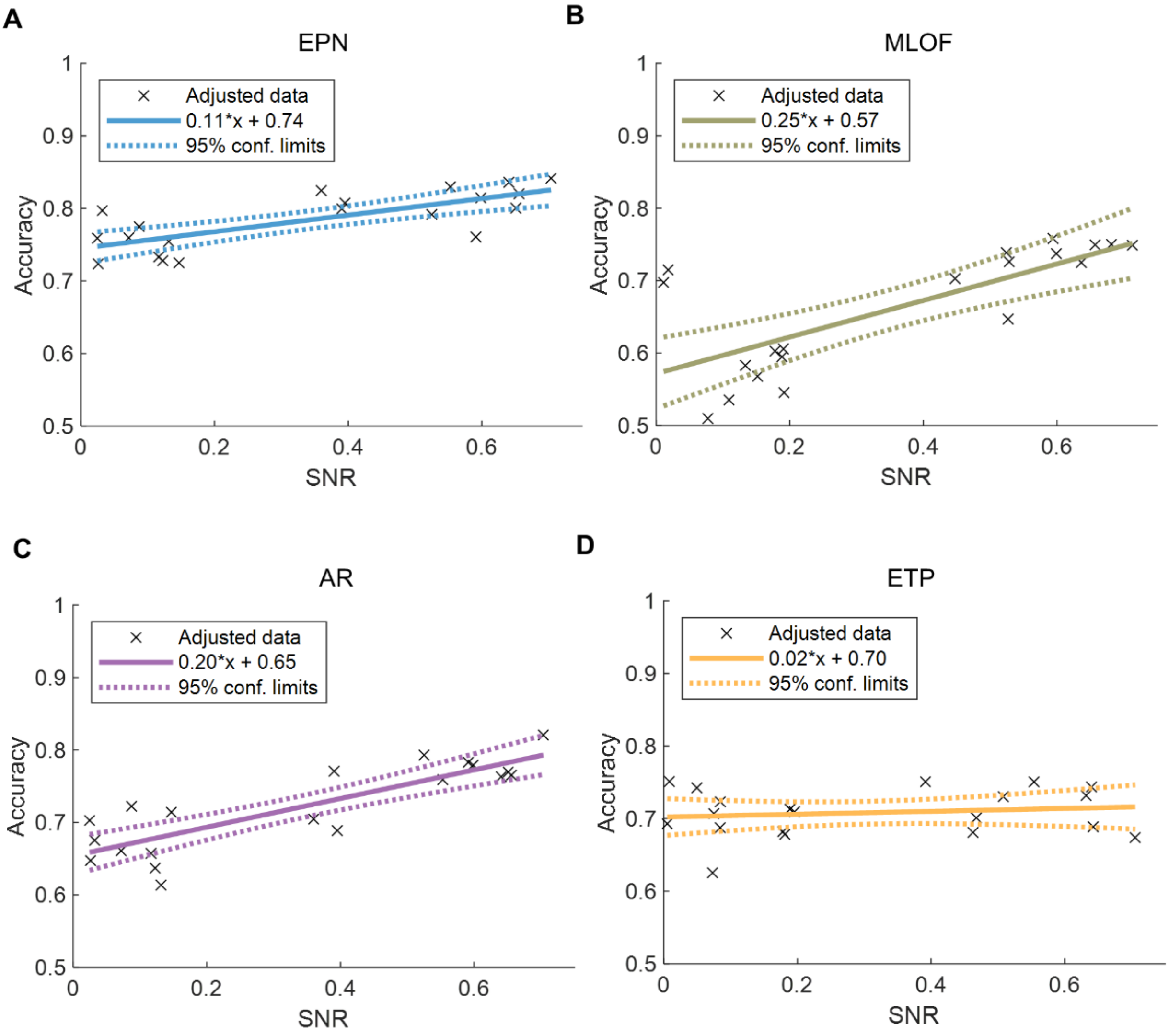

Fig. S5 Dependence between the phase estimation accuracy and the SNR in a real-time experiment. There is a significantly positive relationship between the SNR and the phase estimation accuracy of the EPN (**A**), MLOF (**B**), and AR (**C**) models but the ETP (**D**) model has a non-significant positive relationship. EPN, EEG phase prediction network; MLOF, multi-layer filter architecture; AR, auto-regress; ETP, educated temporal prediction.
